# The Modification of Serum Lipids after Acute Coronary Syndrome and Importance in Clinical Practice 

**DOI:** 10.2174/157340311799960690

**Published:** 2011-11

**Authors:** Bahattin Balci

**Affiliations:** The University of Kafkas, Kars, Turkey

**Keywords:** Acute coronary syndrome, acute phase response, lipid and lipoproteins.

## Abstract

Atherosclerosis is a pathology characterized by low-grade vascular inflammation rather than a mere accumulation of lipids. Inflammation is central at all stages of atherosclerosis. Acute coronary syndrome significantly affects the concentration and composition of the lipids and lipoproteins in plasma. Plasma triglyceride and very low density lipoprotein levels increase, while high density lipoprotein, low density lipoprotein and total cholesterol levels decrease. Early treatment of hyperlipidemia provides potential benefits. However, post-event changes in lipid and lipoproteins lead to delays in the choice of the treatment. This review focuses on the mechanism and the clinical importance of the relevant changes.

## INTRODUCTION

Hyperlipidemia is a major risk factor for coronary heart disease [1]. It is known that treatment of hyperlipidemia reduces the morbidity and mortality of coronary heart disease [2]. Early treatment of hyperlipidemia following acute coronary syndrome (ACS) provides potential benefits [3]. Among patients who have recently experienced an acute coronary syndrome, an intensive lipid-lowering statin regimen provides greater protection against death or major cardiovascular events than does a standard regimen [4]. However, the levels of lipid and lipoproteins change during acute illnesses that causes delays in treatment choice. This review focuses on the changes in the lipids and lipoproteins following ACS and aims to explore the mechanisms and the clinical relevance of these changes. 

## ACUTE PHASE RESPONSE

Inflammation is a process of a series of changes in the area of or away from the inflammation in the case of an inflammatory disease, or such cases as a response to the inflammation and tissue damage of an organism. These changes are common reactions and not specific to a disease, which are called acute phase reactions [5]. Acute phase response includes neuroendocrine, hematopoietic and metabolic changes, including increased cortisol, leukocytosis and enhanced protein catabolism. Acute phase response alters the concentrations of a number of proteins. These proteins are called acute phase proteins. The proteins increasing in amount (such as C-reactive protein, serum amyloid A, ceruloplasmin, complement, haptoglobin, fibrinogen) are called positive acute phase proteins, while proteins decreasing in amount (such as albumin, transferrin, α-2 HS glycoprotein, α-fetoprotein, factor XII) are called negative acute phase proteins. Changes occurring during acute phase are intended to combat against infections and/or to facilitate tissue repair. 

A series of changes in lipid metabolism occurs during acute phase response. As a result, plasma triglyceride (TG) and very low density lipoprotein (VLDL) levels increase, while high density lipoprotein (HDL), low density lipoprotein (LDL) and total cholesterol (TC) levels decrease. It is known that there are changes in lipids and lipoproteins in the course of many disorders characterized by infection and inflammation. For some of the diseases related to the inflammation which causes changes in lipoproteins refer to Table **1**. 

## LIPOPROTEINS IN CONNECTION WITH ACUTE PHASE REACTIONS

Lipoproteins play a significant role in the extracellular transport of lipids. Classification of lipoproteins depends to individual density of molecules, and as follows: Chylomicron (least density), VLDL, LDL, HDL (highest density. General structure of a lipoprotein includes a core consisting of a droplet of triacylglycerols and/or cholesteryl esters and a surface monolayer of phospholipid, unesterified cholesterol and specific proteins.

Structure of LDL is highly heterogeneous, with variances in buoyant density, size, surface charge and chemical composition. [6]. The lipid core of LDL is predominantly composed of cholesteryl esters. LDL contains only one protein, apoprotein B-100, which is associated with the surface monolayer of LDL. There are at least eleven lipid-binding domains. Cells take up LDL by receptor-mediated endocytosis. Lipoproteins containing ApoBs are particularly responsiblee for subendothelial deposition of the LDL via binding to the extracellular matrix molecules with their binding domains [7]. Small, dense LDL subclasses, which are more prone to modification, are more atherogenic than their lighter counterparts. Such modification of LDL occurs primarily through oxidation, enzymatic degradation or lipolysis, are mostly responsible for formation of early atherosclerotic lesions by accumulation of lipids within the arterial wall. The deposition of modified LDL in the arterial subendothelium, where proatherogenic LDL bind to proteoglycans, is a crucial step in the genesis of atherosclerosis. Thereafter, LDL is readily engulfed by macrophages, forming foam cells [6]. 

HDL is not homogenous and rather a mixture of lipoprotein particles. Depending on lipid composition, HDL may have a discoidal or spherical shape. HDL possesses a number of atheroprotective functions. One of these functions is the unloading of excessive cholesterol from peripheral tissues and its transport to the liver for catabolism, a process also known as reverse cholesterol transport. This process includes removal of cholesterol from cholesterol laden peripheral tissues including lipid-laden macrophages which are present at the site of atherosclerotic lesions, and delivery to liver or other cholesterol metabolizing tissues for catabolism. Through this pathway, HDL prevents the excess accumulation of cholesterol within the arterial wall, thus attenuating the progression of atherosclerosis. HDL has antioxidant properties that are mainly due to the presence of apoA-I. HDL provides direct or indirect protection against oxidation of LDL. HDL also protects from inflammation, a major element in the pathogenesis of atherosclerosis. An additional parameter that appears to be related to the atheroprotective functions of HDL is the relative level and distribution of HDL subpopulations in different individuals. [8] Numerous and complex changes arise during acute phase response cause remodelling of HDL [9]. Patients with CHD have reduced α-1, pre α -1, pre-α 2, and pre-α 3 HDL subspecies while their α-3 subspecies appear to be elevated [8]. Therefore, this remodeling creates functional alteratons, including a decrease in cholesterol efflux capacity. HDL inhibits this process by preventing oxidation of the LDL. However, the acute phase response interferes antioxidant properties of HDL, thereby leading to enhanced oxidation of LDL [9]. 

## THE RELATIONSHIP BETWEEN INFLAMMATION AND ATHEROSCLEROSIS

Atherosclerosis is a pathology characterized by low-grade vascular inflammation rather than a mere accumulation of lipids. Finally, chronic inflammatory process involving the arterial endothelium results in the complications of atherosclerosis. Atherosclerosis is a multi-step disease and chronic inflammation play a role in its every stage from its onset, progression and finally to plaque rupture [10]. Many markers of inflammation appear during atherogenesis, which are used for risk prediction, particularly C-reactive protein (CRP) and Serum amyloid-associated protein A. For inflammatory markers that appear with atherosclerosis readers are referred to Table **2**.

Oxidative stress leading to modification of LDL is a central paradigm of atherogenesis and plaque destabilization [11]. The deposition of the LDL into the subendothelial space creates a tendency for LDL to be exposed to oxidation. Under conditions of high oxidant stress, lipoprotein phospholipids become progressively oxidized. These oxidant products cross link and fracture the apolipoprotein B molecule of LDL. Oxidation of the LDL leads to a potent inflammatory response seen in course of atherosclerosis, by causing the synthesis and secretion of monocyte chemoatractan protein-1 in the cellsiniating a stream of biological processes involving atherosclerosis. Primary proinflammatory cytokines (TNF- α, IL-1, IL-18) stimulate the secretion of messenger cytokine IL-6 as well as endothelial adhesion molecules, proteases and other mediators [12]. IL-6 leads to an increase in acute phase reactants, such as CRP liberation from liver. On the other hand, platelets and adipose tissue create inflammatory mediators related to atherosclerosis [13]. Inflammation also acts at the onset of adverse clinical vascular events, when activated cells within the plaque secrete matrix proteases that degrade extracellular matrix proteins and weaken the fibrous cap, leading to rupture and thrombus formation (12)

## THE MODIFICATION OF LIPIDS AND LIPOPROTEINS AFTER ACS

In 1957, Biorck *et al*. first reported that serum cholesterol levels decrease during myocardial infarction [14]. Since then, a wide range of changes in the serum lipid and lipoproteins following ACS have been reported. For rate of changes in lipid and lipoprotein levels after ACS refer to Table **3**. A reduction in the magnitude of these changes is seen over time [15]. In early relevant studies, it was noted that although the levels of TC, LDL and HDL decrease by up to 47%, 39% and 11%, respectively, TG levels rise by up to 50% [16]. There is no consensus with respect to timing of lipid and lipoprotein measurements in terms of proximity to the baseline values; the magnitude of the changes and when these changes reach maximum and basal values. These inconsistent data may be associated with factors such as the changing nature of therapeutic interventions, the retrospective nature of previous studies, studying with relatively small and heterogeneous samples, the use of LDL based on non-fasting blood samples (in this case, the levels of TG would be higher and so the levels of LDL-C would be lower) and the use of different methods (direct and indirect) for measuring the levels of LDL [17]. 

However, the modification of serum lipids includes a decrease in TC, LDL and HDL levels and a reciprocal increase in TG levels. It is thought that these changes become manifest within 24-48 hours after ACS, reach maximum levels within approximately 4-7 days and are present for a few months. Although the changes in the lipids and lipoproteins depend on the extent and the severity of myocardial necrosis and the serum lipid levels, they are not associated with thrombolytic treatment and percutaneous interventions [18]. 

## LIPID MODIFICATION MECHANISMS

Several mechanisms play a role in lipid modification following ACS. For the mechanisms involved in lipid modification refer to Table **4**. Lipid and lipoprotein levels are ascertained after the final effects of acute phase response, adrenergic-mediated adipocyte lipolysis, drugs used during hospitalization, reduction of saturated fat intake and lifestyle changes.

1) Acute phase response: Acute phase response alters not only the concentration of the lipoproteins, but also the composition of the circulating LDL and HDL. The alterations in the level of lipid and lipoproteins are mainly a result of the up-regulation of LDL receptor activity and the decline of proteins involved in the regulation of HDL.

During acute phase reaction, LDL synthesis is increased. LDL particle size is smaller in patients with ACS as compared to non-ACS patients [19]. Despite that, LDL levels decrease due to up-regulation of LDL receptor activity [20]. LDL is not a homogenous particle in plasma and consists of multiple sub-types. Small and dense LDL is more atherogenic [21]. Changes in peak sizes of LDL particles occur in the very early stages of infarction, and a significant increase in size can be detectable 2-3 months after the acute event [22]. In addition, LDL has the predisposition of oxidation exposure following ACS [23].

 Important functions of HDL include reverse transport of cholesterol and modulation of inflammation. Although HDL has anti-inflammatory effects in baseline conditions, it has pro-inflammatory effects during acute phase response [24]. Acute phase response has quantitative and qualitative effects on HDL and its contents. Inflammation decreases the level of HDL by increasing the activity of endothelial lipase and soluble phospholipase A2 and replacing the Apo-A1 in the HDL with serum amyloid A. In addition, significant changes are seen in the protein and lipid composition of HDL [25]. Inflammation leads to changes in the size, composition and function of the HDL. There is a decrease in the levels of several plasma proteins included in HDL-mediated reverse transport of cholesterol and inhibition of lipid oxidation during inflammation. For HDL regulator proteins decreasing during an APR refer to Table **5**. The composition of the circulating HDL changes during an APR, and this is called acute phase HDL. Acute-phase HDL is poor in cholesterol ester, but rich in free cholesterol, TG and free fatty acids. Phospholipid content is variable and may increase or decrease. Apolipoprotein J and serum amyloid A levels increase several-fold. Acute phase HDL is less effective than normal HDL with respect to its protective effects against atherosclerosis [26]. 

Inflammation associated with hypertriglyceridemia is caused by an increase lipoprotein production and a decrease in lipoprotein clearance. Increase in TG-rich lipoproteins is secondary to the re-esterification of plasma fatty acids. Clearance decreases mainly secondary to the inhibition of lipoprotein lipase activity [26].

2) Myocardial damage-induced stress increases the adrenergic-mediated lipolysis of the adipocytes. In conclusion, it leads to an increase in free fatty acids, TGs and lipoproteins [27]. The mobilization of free fatty acids and hepatic secretion of VLDLs increase and lead to the elevation of TG levels.
3) Drugs used in hospital have a role in these modifications. Heparin activates the lipoprotein lipase, so that the internalization of the LDL and VLDL through LDL receptors increases [28]. In conclusion, TG and LDL levels decrease. While non-selective β-blockers increase TG and LDL levels, they decrease the level of HDL [29].
4) Life-style changes and a reduction in saturated fat intake may be effective in reducing the levels of LDL and HDL. While bed rest decreases HDL levels, it increases the level of TG [30].

## THE IMPORTANCE OF LIPID MODIFICATION IN CLINICAL PRACTICE

Although the measurement of serum lipids is recommended after the admission of patients with ACS, serum lipid levels are measured in less than half of the patients. This is important, as the initiation of statin therapy in hospital is related to continuation of treatment after discharge. The idea that there is an early modification in serum lipids and lipoproteins brings the thought that these measurements are not reliable. It was seen that there were phasic changes in serum lipid and lipoprotein levels following an ACS. Therefore, it is difficult to find the patient's baseline lipid profile correctly. However, if we consider that the changes are minimal in the first 24 hours, it seems reasonable to evaluate the lipid profile in that period [31]. On the other hand, it is expected that TC, LDL and HDL levels that are measured following ACS are lower than the basal values. If the resultant value is within the range that requires an intervention on the lipid profile, the baseline values will make the intervention more necessary anyway. 

It is important to measure serum lipids and lipoprotein levels as well as to know their varying characteristics. For example, to measure the level of HDL does not exactly show the composition, functionality, and anti-inflammatory characteristics of HDL. There are no widely-used tests in clinical practice for their measurement. However, these features are directly related to the degree of underlying acute phase response. These should also been taken into account and emphasis should be placed on lifestyle modification, including control of diabetes mellitus, obesity, and hypertension. Inflammation and oxidation play a key role in the pathogenesis of atherosclerosis. Drugs used in current treatment of CAD, namely statins, fibrates, ACE inhibitors and aspirin that are known to have anti-inflammatory characteristics [10]. On the other hand, it has been reported that HDL is dysfunctional in patients with coronary artery disease and acquires similar features with that of healthy individuals following an aggressive lipid therapy [32]. Exact knowledge regarding to serum lipids and lipoprotein levels as well as their varying characteristics may affect decisions on lipid-lowering measures, including necessity of potential supporting lipid treatment.

## CONCLUSION

Lipid modification after ACS should be taken into account and the fasting lipid profile assessment should be performed as soon as possible after admission to the hospital. Increasing evidence suggest that statin therapy reduces morbidity and mortality in patients experiencing an acute coronary event, when initiated immediately after patients' admission. The reduction of LDL following ACS is supported by the evidence based data. Therefore, interventions on the lipid profile of patients diagnosed with ACS should be initiated as soon as possible. In addition, statins should be given to patients with a metabolic status that does not pose a risk, according to guidelines. The fluctuations in lipid and lipoprotein levels should be monitored for a few months after ACS and any clinical decision should be based on them.

## Figures and Tables

**Table 1.** Some of the Diseases Related to the Inflammation which Causes Changes in Lipoproteins

Rheumatic Diseases
Infectious diseases
Cystic fibrosis
Diabetes Mellitus
Chronic renal failure
Obesity
Acute myocardial infarction

**Table 2.** The Inflammatory Markers that Appear with Atherosclerosis

C reactive protein
Fibrinogen
Serum amyloid A
Plasminogen activator inhibitor-1
Adhesion molecules
Cytokines
Reactive oxygen species
Matrix metalloproteinases
Adiponectin

**Table 3.** Rate of Changes in Lipid and Lipoprotein Levels after ACS

TC	-1.25% to -47%
LDL	-1.7% to -39%
HDL	0.0% to -11%
TG	+9.8% to +50%

TC: Total cholesterol, LDL: Low-density cholesterol, HDL: High-density cholesterol, TG: Triglyceride

**Table 4.** The Mechanisms Involved in Lipid Modification

Acute phase response
Adrenergic-mediated adipocyte lipolysis
The drugs used during hospitalization
The reduction of saturated fat intake, lifestyle changes

**Table 5.** HDL Regulator Proteins Decreasing During an APR

Lecithin-cholestero acyltransferase (LCAT)
Cholesterol ester transfer protein
Phospholipid transfer protein
Hepatic lipase
Apolipoprotein A-I
Paraoxonase
